# Dexmedetomidine Leads to the Mitigation of Myocardial Ischemia/Reperfusion-Induced Acute Lung Injury in Diabetic Rats Via Modulation of Hypoxia-Inducible Factor-1α

**DOI:** 10.21470/1678-9741-2020-0591

**Published:** 2022

**Authors:** Siyu Chen, Jianjiang Wu, Long Yang, Taiwangu Tailaiti, Tiantian Zou, Yidan Huan, Jiang Wang

**Affiliations:** 1 The First Affiliated Hospital of Xinjiang Medical University, Urumqi, Xinjiang Uygur Autonomous Region, Urumqi, People’s Republic of China.

**Keywords:** Dexmedetomidine, Acute Lung Injury, Diabetes, Myocardial Ischemia Reperfusion, Hypoxia-Inducible Factor 1, Up-Regulation

## Abstract

**Introduction:**

The objective of this study is to investigate the protective mechanism of dexmedetomidine (Dex) in myocardial ischemia/reperfusion (MIR)-induced acute lung injury (ALI) of diabetic rats by inhibiting hypoxia-inducible factor-1α (HIF-1α).

**Methods:**

Initially, healthy male Sprague Dawley rats were treated with streptozocin to induce diabetes. Then, three weeks after the induction, Dex or lentiviral vector (LV)-HIF-1α was injected into the rats 30 minutes prior to the MIR modeling. After four weeks, lung tissues were harvested for pathological changes observation and the wet/dry weight (W/D) ratio determination. Afterwards, oxidative stress indicators and pro-inflammatory factors were measured. In addition, HIF-1α expression was assessed by immunohistochemistry and western blot analysis.

**Results:**

Dex could suppress inflammatory cell infiltration, improve lung tissue structure, reduce pathological score and the W/D ratio, and block oxidative stress and inflammatory response in MIR-induced ALI of diabetic rats. Besides, Dex could also inhibit HIF-1α expression. Moreover, Dex + LV-HIF-1α reversed the protective role of Dex on diabetic MIR-induced ALI.

**Conclusion:**

Our study has made it clear that Dex inhibited the upregulation of HIF-1α in diabetic MIR-induced ALI, and thus protect lung functions by quenching the accumulation of oxygen radical and reducing lung inflammatory response.

**Table:** 

Abbreviations, acronyms & symbols			
ALI	= Acute lung injury	LVDP	= Left ventricular developed pressure
ANOVA	= Analysis of variance	MDA	= Malondialdehyde
D	= Dry weight	MI	= Myocardial ischemia
Dex	= Dexmedetomidine	MIR	= Myocardial ischemia/reperfusion
DIR	= Diabetic + IR	MIRI	= Myocardial ischemia/reperfusion injury
DS	= Diabetic sham	NC	= Negative control
ELISA	= Enzyme-linked immunosorbent assay	NIR	= Non-diabetic + IR
HE	= Hematoxylin and eosin	NO	= Nitric oxide
HIF-1α	= Hypoxia-inducible factor-1	NS	= Non-diabetic sham
HRP	= Horseradish peroxidase	ROS	= Reactive oxygen species
IgG	= Immunoglobulin G	SOD	= Superoxide dismutase
IHC	= Immunohistochemistry	STZ	= Streptozocin
IL	= Interleukin	TNF-α	= Tumor necrosis factor-α
IP	= Intraperitoneal	TTC	= 2, 3, 5-triphenyltetrazolium chloride
IR	= Ischemia/reperfusion	USA	= United States of America
IRI	= Ischemia/reperfusion injury	W	= Wet weight
LI	= Lung injury	W/D	= Wet/dry weight
LV	= Lentiviral vector		

## INTRODUCTION

Diabetes is a series of physiological disorders triggered by insufficient insulin release, overproduction of glucagon, and insulin resistance^[1]^. In a great number of diabetic patients, cardiovascular dysfunction may be the most common complication that leads to numerous injuries and death^[2]^. The features that increase oxidative stress and impair antioxidant ability in diabetic hearts make them vulnerable to myocardial ischemia/reperfusion injury (MIRI)^[3]^. MIRI refers to a pathological progress of myocardial damage caused by blood flow restoration after ischemia that affects some organs^[4]^. Overproduction of free radicals and reactive oxygen species (ROS) is identified as the leading cause of the pathogenesis of ischemia/reperfusion injury (IRI)^[5]^. Inflammatory factor cascade induces secondary injury, exacerbating IRI^[6]^. Diabetic individuals that are susceptible to MIRI are likely to develop acute lung injury (ALI) for the excessive inflammatory response and ROS integration^[7]^. Besides, as some previous experiments have suggested, diseases derived from diabetes are often related to lipid peroxidation, which is frequently seen in the process during ischemia/reperfusion (IR), and it exacerbates the functions of many distal organs, including lung^[8,9]^. In this context, novel therapeutic strategies for myocardial ischemia/reperfusion (MIR)-induced ALI in diabetic patients are in urgent need. Therefore, our study was designed to seek out the roles of different pathological mechanisms playing in diabetic MIR-induced ALI in order to develop novel intervention strategies.

Dexmedetomidine (Dex) is an α_2_-adrenoceptor agonist with high selection, which has opioid-sparing, analgesic, and sedative effects, ensuring it a potent target in treating intensive diseases^[10]^. In a previous research, Dex was indicated to render a cardioprotective function on heart tissues in diabetic rats by preventing against inflammatory reaction^[11]^. It has also been reported by Kip et al. that Dex is a beneficial treatment of lung injury (LI) caused by MIR in diabetic rats^[9]^. Although the mechanism of Dex in diabetes or MIR-derived diseases is widely studied, there are few researches about the role of Dex in diabetic MIR-induced ALI, let alone the specific crosswalk between Dex and other genes. Besides, inflammatory reaction and oxidative stress are remarkable signs of ALI with the presentation of increased hypoxia-inducible factor-1α (HIF-1α) level^[12,13]^. As a highly conserved transcription cytokine, HIF-1α is responsible for the hypoxia reaction and inflammatory response in hearts^[14]^. Moreover, dysregulation of HIF-1α pathway is a necessary mechanism in MIRI of diabetic rats^[15]^. Similarly, in cerebral IRI-induced ALI, HIF-1α overexpression is observed, indicating the impaired endothelial barrier and weakened self-protective ability^[16]^. Peng et al.^[17]^ has demonstrated that Dex inhibits HIF-1α expression to relieve cardiac IRI. In this sense, we are encouraged to hypothesize that the network of Dex and HIF-1α might be importantly involved in the treatment of MIR-induced ALI in diabetic rats.

## METHODS

### Ethics Statement

This study was approved and supervised by the ethics committee of our hospital (IACUC-20190225-23). Significant efforts were made to minimize both the number of animals used as well as their respective suffering.

### Animal Grouping

**Fig. 1:**
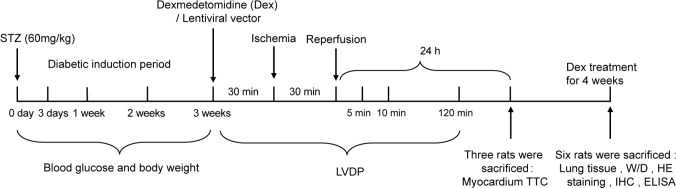
Timeline of the treatment of the studied rats. The figure shows the diabetes induction period, ischemia/reperfusion period, Dex administration time points, animal sacrifice time points, and test indicators of each time point. ELISA=enzyme-linked immunosorbent assay; HE=hematoxylin and eosin; IHC=immunohistochemistry; LVDP=left ventricular developed pressure; STZ=streptozocin; TTC=2, 3, 5-triphenyltetrazolium chloride; W/D=wet/dry weight.

A total of 63 Sprague Dawley rats (250-300 g, eight weeks) (Hunan SJA Laboratory Animal Co., Ltd., Changsha, Hunan, China) were housed in the laboratory animal center of our hospital, with a normal circadian rhythm (12-hour light/dark cycle) and a relative humidity of 50%±15% at 25±2℃. Standard food and water were available to them. These rats were randomly assigned into non-diabetic sham (NS) group, non-diabetic + IR (NIR) group, diabetic sham (DS) group, diabetic + IR (DIR) group, Dex group, Dex + lentiviral vector (LV)-negative control (NC) group, and Dex + LV-HIF-1α group (n=9). The timeline of the treatment of these rats is shown in Figure 1.

### Streptozocin (STZ)-Induced Diabetes

Diabetes was induced through the intraperitoneal (IP) injection of STZ (Sigma-Aldrich, Merck KGaA, Darmstadt, Germany). STZ was dissolved in citrate buffer (pH 4.5) and was applied via IP injection at 60 mg/kg^[7]^. NS and NIR groups were injected with buffer only (10 mM citric buffer, pH 4.5) as control after the same fasting period. After three days, one week, two weeks, and three weeks of STZ injection, tail blood samples were collected after rats had fasted for five hours. Fasting blood glucose level was measured using a SureStrep glucometer (Johnson & Johnson, New Brunswick, New Jersey, United States of America [USA]). A fasting blood glucose persistently exceeding 16.7 mmol/L was recognized as diabetic status^[18]^.

### MIR Model Establishment

Rats were anesthetized with IP injection of 3% sodium pentobarbital (50 mg/kg) (Sigma-Aldrich, USA) three weeks after the initial administration of STZ or control for the following left thoracotomy and pericardiotomy. The left coronary artery was dissected above the first diagonal branch. Then, threads were ligated with silk sutures at the proximal origin of the left circumflex artery. Once the gliding-induced occlusion was performed for 30 minutes, R-wave amplification and ST-segment depression were observed on the second lead of the attached electrocardiogram. Myocardial darkening distal to the ligature suggested myocardial ischemia (MI). After the 30-minute MI, slipknot was released for 120 min to allow reperfusion^[19]^. In the Dex group, 7.5 µg/kg/h of Dex was injected intravenously at a rate of 5 µg/kg/h 30 minutes before modeling, and the other groups were injected with the same amount of saline^[20,21]^. LV-NC or LV-HIF-1α (Shanghai Genechem Co., Ltd., Shanghai, China, 5.25×10^7^ transducing units, and a total volume of 25 mL) was injected into the free myocardium and apex of left ventricular anterior wall on both sides of the left coronary artery 30 minutes before modeling^[22]^.

### Hemodynamics and 2, 3, 5-Triphenyltetrazolium Chloride (TTC) Staining

Mikro Tip® catheter pressure transducer (BL420F Powerlab, Taimong Technology Co., Ltd., Hong Kong, China) was inserted into the left ventricular chamber via the right common carotid artery to monitor the changes in left ventricular developed pressure (LVDP). Data were continuously recorded at the beginning of reperfusion. After 24 hours of reperfusion, three rats from each group were sacrificed to remove their hearts, and the myocardial infarct’s size was measured via 1% TTC staining according to a previous experiment^[23]^. Myocardial infarct’s size was expressed as the percentage of the risk area.

### Lung Tissue Collection

After four weeks of Dex treatment, rats were sacrificed with their upper lobes of the right lung separated for pathological examination and protein determination. Wet weight (W) was measured as the left lungs were flushed with cold isotonic saline and then blotted with filter paper. Additionally, the right lungs were placed in a 60℃ furnace for 72 hours to assess the dry weight (D). Subsequently, the W/D ratio was calculated.

### Hematoxylin and Eosin (HE) Staining

The upper lobes of the right lung of rats were fixed in 10% formalin overnight and were made into paraffin-embedded sections (4-mm thick) for the preparation of HE staining. LI was assessed by a designated scoring system that quantified the degree of histological LI, with four histological findings used for grading: hemorrhage; alveolar congestion; filtration or accumulation of neutrophils in airspace or vessel wall; and thickness between alveolar wall and hyaline membrane. The severity of inflammation ranged from 0 to 4^[7]^. LI score was measured by two experienced pathologists who were blinded to the samples.

### Immunohistochemistry (IHC) Staining

All paraffin-embedded tissues were dewaxed, treated with 10 mM sodium citrate for heat-induced antigen recovery, incubated with 0.3% H_2_O_2_ for 20 minutes at room temperature, and then co-cultured with 20% normal goat serum for 30 minutes. Immunohistochemistry was performed using mouse anti-HIF-1α monoclonal antibody (Santa Cruz Biotechnology, Dallas, Texas, USA) diluted at 1:100 in blocking reagent at 4℃ overnight. The slides were continuously cultivated with 1:200 horseradish peroxidase (HRP)-conjugated goat anti-mouse immunoglobulin G (IgG) (Chemicon International, Inc., Temecula, California, USA) for two hours at room temperature. They were then developed with diaminobenzidine for 8-12 minutes, counterstained with hematoxylin, dried, and then fixed with resin. The images were obtained using a cell image analysis system and medical image analysis software, and the data were quantitatively analyzed. Three separate visual fields were randomly selected from each slice to obtain the percentage of positive area in each sample (positive cell area/total area) and to be used for analysis. The results were assessed by two experienced pathologists who were blinded to the samples.

### Western Blot Analysis

The total protein was extracted from lung tissue homogenate, and then quantified using bicinchoninic acid method. Next, proteins were run on sodium dodecyl sulfate polyacrylamide gel electrophoresis and then transferred onto polyvinylidene fluoride membranes (Millipore Corp., Billerica, Massachusetts, USA). The membranes were sealed by 5% skim milk powder for one hour and cultured with the primary antibodies, including β-actin and HIF-1α, at 4℃ overnight. After one rinse with poly (butylene succinate-terephthalate), membranes were incubated with HRP-labelled IgG (1:10000) at room temperature for one hour. Following the instructions of chemiluminescence (Millipore), blots were observed. Afterwards, relative band intensity was quantified using image lab analyzer software (Bio-Rad, Hercules, California, USA). Eventually, the ratio of the gray value of the target band to internal reference band was used as the relative targeted protein expression.

### Detection of Superoxide Dismutase (SOD), Malondialdehyde (MDA), and Nitric Oxide (NO)

Lung tissues were taken, made into 10% tissue homogenate in cold normal saline, and centrifuged with supernatant separately. Next, levels of SOD, MDA, and NO were verified according to the instructions of the kits (Nanjing Jiancheng Bioengineering Institute, Nanjing, Jiangsu, China).

### Enzyme-Linked Immunosorbent Assay (ELISA)

Levels of pro-inflammatory cytokines including tumor necrosis factor-α (TNF-α), interleukin (IL)-1β, and IL-6 in lung tissue homogenate were assessed in accordance with the instructions of ELISA kits (R&D Systems, Minneapolis, Minnesota, USA).

### Statistical Analysis

GraphPad Prism 8.0 (GraphPad Software, La Jolla, California, USA) was employed for data analysis. The results were showed in mean ± standard deviation. The *t*-test was applied for analysis of comparisons between two groups. One-way analysis of variance (ANOVA) was applied for comparing different groups, and Tukey's multiple comparisons test for pairwise comparisons after ANOVA. Repeated ANOVA was performed for comparing different time points. The *P*-value was obtained using a two-tailed test and *P*<0.05 indicated a significant difference.

## RESULTS

### Identification of MIRI in Diabetic Mouse Models

**Fig. 2:**
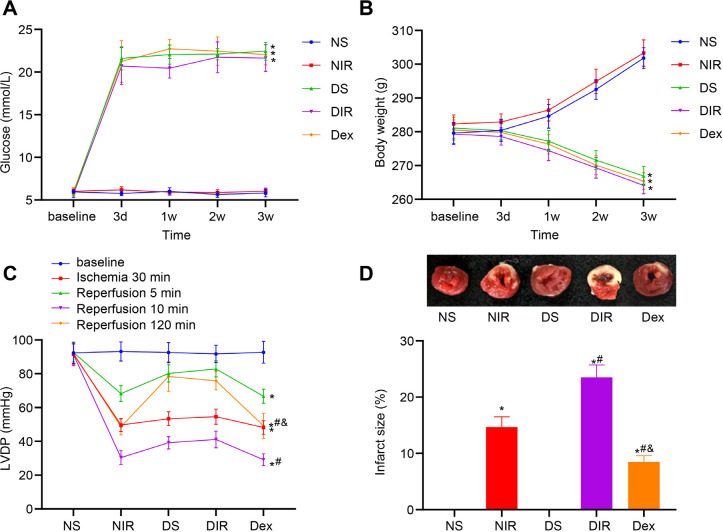
Identification of myocardial ischemia/reperfusion (MIR) injury in diabetic mouse models. A-B) Glucose (A) and body weights (B) of diabetic rats at three days, one week, two weeks, and three weeks after streptozocin induction. C) Left ventricular developed pressure (LVDP) level in each group after MIR (n=6). D) Infarct’s size of each group after MIR observed by 2, 3, 5-triphenyltetrazolium chloride staining (n=3). One-way analysis of variance (ANOVA) and repeated ANOVA were employed to verify statistical significance. * P<0.05, compared with the non-diabetic sham (NS) group; # P<0.05, compared with the diabetic sham (DS) group; & P<0.05, compared with the diabetic + ischemia/reperfusion (DIR) group. Dex=dexmedetomidine; NIR=non-diabetic + ischemia/reperfusion.

Diabetic rat models were established by STZ induction. At the end of our experiment, blood glucose and body weight loss were significantly enhanced in STZ-induced diabetic rats compared with non-diabetic rats (all *P*<0.05, Figures 2A and B), suggesting the successfully established diabetic modeling. After MIR in rats, all groups exhibited dramatically reduced LVDP, except the NS group, and the DIR group showed the most significant decrease (all *P*<0.05, Figure 2C). TTC staining (Figure 2D) showed that compared with the NS group, the infarct’s size of the NIR, DIR, and Dex groups was noticeably expanded, and the DIR group expressed the most significant increase (all *P*<0.05); on the other hand, the infarct’s size of the Dex group was smaller than that of the DIR group (all *P*<0.05). These results suggested that diabetic rats were susceptible to MIRI, which could be attenuated by Dex.

### Dex Relieves Diabetic MIR-Induced ALI

**Fig. 3:**
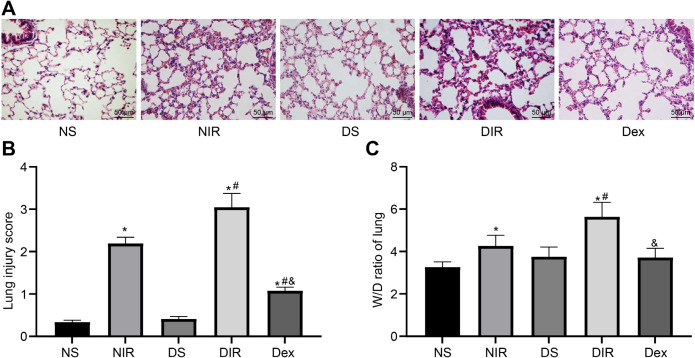
Dexmedetomidine (Dex) relieves diabetic myocardial ischemia/reperfusion-induced acute lung injury. A-C) Representative hematoxylin and eosin staining images (A), pathological injury score (B), and wet/dry weight (W/D) ratio (C) of lung tissue sections from rats treated with Dex for four weeks (×200) (n=6). One-way analysis of variance was employed to verify statistical significance. *P<0.05, compared with the non-diabetic sham (NS) group; #P<0.05, compared with the diabetic sham (DS) group; & P<0.05, compared with the diabetic + ischemia/reperfusion (DIR) group. NIR=non-diabetic + ischemia/reperfusion.

HE pathological staining was applied to analyze lung tissues from the rats subjected to Dex treatment for four weeks to verify the protective role of Dex on diabetic MIR-induced ALI, and the results showed that there were no significant pathological changes in the lung tissue sections from the NS and DS groups. Besides, alveolar and interstitial edema, hemorrhage, inflammatory cell infiltration, and elevated pathological score were observed in lung sections from the NIR group. However, these indicators were even more advanced in the DIR group. While in the Dex group, inflammatory cell infiltration was significantly reduced, lung tissue structure was improved, and pathological score was decreased (Figures 3A and B). The W/D ratio in the NIR and DIR groups was higher than that in the NS and DS groups (all *P*<0.05); while the W/D ratio in the Dex group was lower than that in the DIR group (*P*<0.05) (Figure 3C), indicating that diabetic MIR might induce ALI, which could be reversed by Dex.

### Dex Suppresses Oxidative Stress (Oxygen Radical) and Inflammatory Response in Diabetic MIR-Induced ALI

**Fig. 4:**
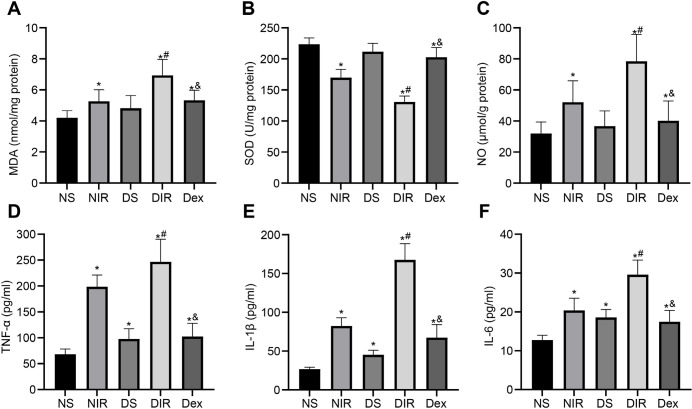
Dexmedetomidine (Dex) suppresses oxidative stress (oxygen radical) and inflammatory response in diabetic myocardial ischemia/reperfusion-induced acute lung injury. A-C) Levels of oxidative stress indicators including malondialdehyde (MDA) (A), superoxide dismutase (SOD) (B), and nitric oxide (NO) (C) in lung tissues from each group after being treated by Dex for four weeks. D-F) Levels of pro-inflammatory factors including tumor necrosis factor-α (TNF-α) (D), interleukin (IL)-1β (E), and IL-6 (F) in lung tissues from each group after being treated with Dex for four weeks (n=6). One-way analysis of variance was employed to verify statistical significance. *P<0.05, compared with the non-diabetic sham (NS) group; #P<0.05, compared with the diabetic sham (DS) group; & P<0.05, compared with the diabetic + ischemia/reperfusion (DIR) group. NIR=non-diabetic + ischemia/reperfusion.

Next, we further determined the protective function of Dex on diabetic MIR-induced ALI by analyzing the effects of Dex on oxidative stress and inflammatory response. The results showed that there was no significant difference in levels of oxidative stress indicators (MDA, SOD, and NO) between lung tissues of the NS and DS groups; levels of MDA and NO were increased, but SOD was evidently decreased after MIR (all *P*<0.05). On the other hand, in the Dex group, MDA and NO levels were significantly reduced, but SOD level was elevated (all *P*<0.05) (Figures 4A to C). Compared with the NS group, the levels of pro-inflammatory factors (TNF-α, IL-1β, and IL-6) in the lung tissues of rats from the other groups were significantly upregulated (all *P*<0.05); and the levels of TNF-α, IL-1β, and IL-6 in the DIR group was further increased (all *P*<0.05); but the Dex group contributed to a reversed result (all *P*<0.05) (Figures 4D to F). That’s to say, Dex blocked oxidative stress and inflammatory responses of MIR-induced ALI in diabetic rats.

### Dex Inhibits HIF-1α Expression in Lung Tissues from the Diabetic MIR-Induced ALI Rats

**Fig. 5:**
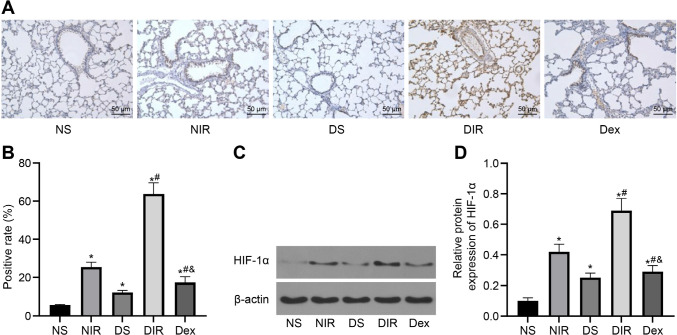
Dexmedetomidine (Dex) inhibits hypoxia-inducible factor-1α (HIF-1α) in lung tissues from the diabetic myocardial ischemia/reperfusion-induced acute lung injury rats. A-B) HIF-1α expression (A) and positive rate (B) in lung tissues from each group after being treated by Dex for four weeks examined by immunohistochemistry. C-D) HIF-1α expression (C) and grey value (D) in lung tissues from each group after being treated by Dex for four weeks tested by western blot analysis. *P<0.05, compared with the non-diabetic sham (NS) group;^#^P<0.05, compared with the diabetic sham (DS) group; & P<0.05, compared with the diabetic + ischemia/reperfusion (DIR) group. NIR=non-diabetic + ischemia/reperfusion.

HIF-1α is an accepted player in diabetic MIR and ALI alleviation^[13,15]^. Based on this information, our study attempted to determine whether Dex could affect HIF-1α expression in diabetic MIR rats, thus immunohistochemistry (Figures 5A and B) and western blot analysis (Figures 5C and D) were conducted to detect HIF-1α expression, and the results showed that the expression of HIF-1α was upregulated in lung tissues of rats with diabetic and MIR treatments, and the highest HIF-1α expression was seen in the diabetic MIR rats; while the upregulation of HIF-1α expression in diabetic MIR rats was reduced after the four-week Dex treatment (all *P*<0.05). The abovementioned results indicated that Dex deactivated HIF-1α in the diabetic MIR-induced ALI rats.

### Dex Attenuates Diabetic MIR-Induced ALI by Limiting HIF-1α

**Fig. 6:**
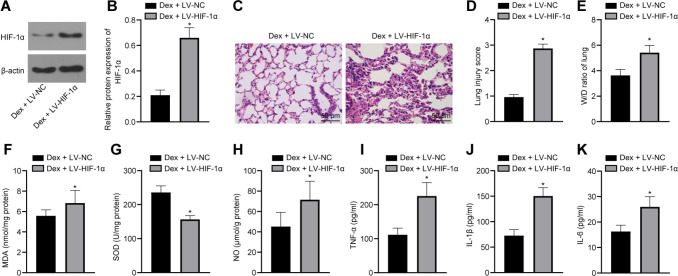
Dexmedetomidine (Dex) attenuates diabetic myocardial ischemia/reperfusion-induced acute lung injury by limiting hypoxia-inducible factor-1α (HIF-1α). A-B) HIF-1α expression (A) and grey value (B) in lung tissues from each group after being treated by Dex + lentiviral vector (LV)-HIF-1α for four weeks detected by western blot analysis. C-E) Representative hematoxylin and eosin staining images (×200) (C), pathological score (D), and the wet/dry weight (W/D) ratio in lung tissues from each group after being treated with Dex + LV-HIF-1α for four weeks. F-H) Levels of oxidative stress indicators including malondialdehyde (MDA) (F), superoxide dismutase (SOD) (G), and nitric oxide (NO) (H) in lung tissues from each group after being treated with Dex + LV-HIF-1α for four weeks. I-K) Tumor necrosis factor-α (TNF-α) (I), interleukin (IL)-1β (J), and IL-6 (K) in lung tissues from each group after being treated with Dex + LV-HIF-1α for four weeks (n=6). The t-test was employed to verify statistical significance. *P<0.05, compared with the Dex + LV-negative control (NC) group. DIR=diabetic + ischemia/reperfusion; DS=diabetic sham; NIR=non-diabetic + ischemia/reperfusion; NS=non-diabetic sham.

Finally, to further verify whether Dex could attenuate MIR-induced ALI by inhibiting HIF-1α, we injected LV-NC or LV-HIF-1α while administrating Dex, and we found that the Dex + LV-HIF-1α group exhibited upregulated HIF-1α expression in lung tissue (Figures 6A and B), alveolar and interstitial edema, hemorrhage, and inflammatory cell infiltration in lung sections, improved pathological score (Figures 6C and D), and increased W/D ratio (Figure 6E). Then, it was revealed that the Dex + LV-HIF-1α group had elevated levels of MDA and NO, but reduced SOD level (all *P*<0.05) (Figures 6F to H), as well as promoted levels of TNF-α, IL-1β, and IL-6 (all *P*<0.05) (Figures 6I to L) as we further detect the effects of HIF-1α on the cytokines of oxidative stress and inflammatory response in lung homogenate. It can be concluded that the upregulation of HIF-1α expression could reverse the oppressive function of Dex on oxidative stress and inflammatory response in MIR-induced ALI.

## DISCUSSION

Diabetic patients are strongly inclined to suffer from MIR, which appears to be a primary reason of distal organ dysfunctions, with lung being the most fragile part^[24]^. As a novel class of medicine with sedative ability, analgesic capacity, and reliable safety, Dex had a promising prospect in renoprotection and myocardial disease clinical therapy^[25]^. For instance, Dex potentiated the self-protective effects of diabetic rats with MIRI^[21]^. In addition, increasing evidences have corroborated the conducive role of Dex in ALI induced by different pathogenesis^[26-28]^. Therefore, we were inspired to figure out the concrete interaction of Dex in diabetic rats with MIR-induced ALI. And we verified that Dex suppressed diabetic MIR-induced ALI by downregulating HIF-1α.

Initially, this study revealed that Dex could alleviate MIRI in diabetic rats. Dex strengthened heart function in diabetic rats as it protected these rats against inflammation and oxidative stress^[29]^. Data had supported that Dex could significantly prevent various animal models from IRI by regulating cell death, gene expression, inflammatory response, and pathway activation, making it an attractive implication in IR-induced dysfunctions^[30]^. Furthermore, Dex could quench lung edema and pathological damage score in lung IRI^[31]^. In other words, Dex reduced the detrimental influences resulted from IR in diabetes. Subsequently, Dex inhibited MIR-induced ALI in diabetic rats. Lung was identified as an important target part in diabetes as disordered respiratory functions were prominent in diabetic subjects^[32]^. Also, Dex administration reversed pathological variances and histological LI score, and thus mitigated other distant organ damage via the inhibition of inflammation^[33]^. Generally speaking, Dex could also protect individuals with ALI. Notably, Dex was a potent inhibitor of oxidative stress and inflammatory response of MIR-induced ALI in diabetic rats as evidenced by the knockdown of MDA and NO, increased SOD level, and reduced levels of TNF-α, IL-1β, and IL-6. It was known to us that oxidative stress and inflammatory reaction were both involved in the incidence of ALI in diabetes^[34]^. A prior experiment suggested that Dex treatment upregulated SOD expression, while deactivated MDA activity^[35]^. Besides, the pro-inflammatory cytokines including TNF-α, IL-6, and IL-1β were all downregulated by Dex administration^[36]^. To sum up, Dex was a significant participator in the inhibition of MIR-induced ALI in diabetic rats through suppressing inflammatory reaction and oxidative stress.

Next, according to the results of immunohistochemistry and western blot analysis, it was unveiled that Dex suppressed HIF-1α expression in lung tissues from the diabetic MIR-induced ALI rats. As a pivotal modulator in hypoxia, which was positively and closely related to inflammatory response, HIF-1α was a hot topic of multiple human diseases^[37]^. Hypoxia participated in different physiological disorders, such as IRI, diabetic vasculopathy, and even cancer through the manipulation of HIF-1α-related pathways^[38]^. Furthermore, inactivation of HIF-1α was a practical method to block MIRI in rats^[17]^. An experiment developed from Cahayani et al.^[39]^ suggested that HIF-1α was strongly expressed in mice with malaria-induced ALI. On the other hand, HIF-1α knockout in *Escherichia coli*-induced ALI limited inflammatory cell infiltration, lung infection, and edema^[40]^. Basically, it was quite clear that HIF-1α could deteriorate MIR-induced ALI in diabetic rats. Afterwards, many researches indicated that Dex protected IR rats by quenching HIF-1α activity^[41,42]^. Therefore, we discovered that the interaction between Dex and HIF-1α is essential in IRI. Additionally, we discovered that HIF-1α reversed the protective function of Dex on diabetic MIR-induced ALI, with increase of MDA and NO levels, decrease of SOD level, and promotion of TNF-α, IL-1β, and IL-6 levels. In gastric IRI condition, promotion of HIF-1α content was coupled with increased MDA and decreased SOD^[43]^. Besides, HIF-1α depletion limited TNF-α and IL-1β content, thus alleviating intestinal IRI and subsequent IRI-induced ALI^[44]^.

### Limitations

In this paper, the protective role of Dex in MIR-induced ALI in diabetic rats by inhibiting HIF-1α was only verified through *in vivo* experiment, but the specific mechanism of Dex inhibiting HIF-1α expression needs to be further investigated.

## CONCLUSION

In conclusion, our findings supported that Dex inhibited the upregulation of HIF-1α in diabetic MIR-induced ALI. These results discovered an underlying tactic for MIR-induced ALI treatment in diabetic patients. In the future, we will continue to explore the underlying mechanism in the disease and the potential therapeutic usage. Currently, this still remains a preclinical research. However, our findings provide therapeutic approaches in diabetic MIR-induced ALI treatment; the experiment results and effective application into clinical practice need further validation. It still has a long way to go before it is widely applied in clinical practice.

**Table:** 

Authors' roles & responsibilities	
SC	Substantial contributions to the conception or design of the work; or the acquisition, analysis, or interpretation of data for the work; drafting the work or revising it critically for important intellectual content; final approval of the version to be published
JW	Substantial contributions to the conception or design of the work; or the acquisition, analysis, or interpretation of data for the work; drafting the work or revising it critically for important intellectual content; final approval of the version to be published
LY	Substantial contributions to the conception or design of the work; or the acquisition, analysis, or interpretation of data for the work; drafting the work or revising it critically for important intellectual content; final approval of the version to be published
TT	Substantial contributions to the conception or design of the work; or the acquisition, analysis, or interpretation of data for the work; drafting the work or revising it critically for important intellectual content; final approval of the version to be published
TZ	Substantial contributions to the conception or design of the work; or the acquisition, analysis, or interpretation of data for the work; drafting the work or revising it critically for important intellectual content; final approval of the version to be published
YH	Substantial contributions to the conception or design of the work; or the acquisition, analysis, or interpretation of data for the work; drafting the work or revising it critically for important intellectual content; final approval of the version to be published
JW	Substantial contributions to the conception or design of the work; or the acquisition, analysis, or interpretation of data for the work; drafting the work or revising it critically for important intellectual content; final approval of the version to be published
